# Abscopal Effect of Radiotherapy in the Immunotherapy Era: Systematic Review of Reported Cases

**DOI:** 10.7759/cureus.4103

**Published:** 2019-02-20

**Authors:** Nergiz Dagoglu, Sule Karaman, Hale B Caglar, Ethem N Oral

**Affiliations:** 1 Radiation Oncology, Istanbul University Faculty of Medicine, Istanbul, TUR; 2 Radiation Oncology, Anadolu Medical Center, Kocaeli, TUR

**Keywords:** radiotherapy, abscopal effect, immunotherapy, immune modulation

## Abstract

Mounting evidence suggests that radiation stimulates the immune system and this contributes to the abscopal effect, which is defined as “response at a distance from the irradiated volume.” Though identified more than 50 years ago, the abscopal effect is revisited today. One rationale is that the abscopal effect is often observed with efficient immunotherapy. Here, we give an overview of the clinical data on the abscopal effect, generated by a combination of immunotherapy and radiotherapy (RT). Only papers that included RT in combination with immunotherapy were evaluated according to four main categories including RT parameters, sequencing of therapies, the definition of the abscopal effect, and patient selection. Twenty-four cases in 15 reports were reviewed. The results varied. Patient ages ranged from 24 to 74. RT dose (median total dose 18-58 Gy) varied. Biologically effective dose (BED) 10 was calculated to be a median 49.65 Gy (28-151 Gy). The time to a documented abscopal response ranged from less than a month to 12 months. The large variation concerning fractionation and sequencing of therapies indicates that these conflicting points need to be resolved, to generate for the abscopal effect to be clinically significant.

## Introduction and background

Radiotherapy (RT) has been used as an effective local treatment modality in cancer management for decades. The therapeutic effect of irradiation is principally thought to come from the deoxyribonucleic acid (DNA) damage that affects the rapidly proliferating cells like cancer cells. However, preclinical studies additionally substantiate that the radiation stimulates the immune system and that radiation is capable of inducing tumor-specific immunity or immunogenic cell death [1]. As a result of this synergy, an effect is described at distant points, which is known as the abscopal effect. The origin of this term is a combination of the Latin root words “ab-", meaning “far,” and “-scopus,” meaning “target” [2]. Although this phenomenon was first described by Mole in 1953, it only recently garnered revived clinical attention [3]. One theory behind this is the recent developments in immunotherapy as there is a growing consensus that it would be easier to obtain an abscopal effect with immunotherapies and immunomodulation.

Today, many clinical trials are reported as ongoing or planned investigating the use of RT with immunotherapy. However, the optimal combinations to generate an abscopal effect are unclear. Four main questions remain to be answered in optimal trial design and evaluation, including RT parameters, the sequencing of therapies, the definition of the abscopal effect, and patient selection [4].

Here, we overview case reports including clinical experience regarding an abscopal effect, to summarize the patient and treatment characteristics, that may lead to the abscopal effect.

Search strategy and selection criteria

Cases to be included in this review were identified through a PubMed search, using the terms “abscopal,” “(non-targeted irradiation) or (non-targeted radiotherapy),” and “distant bystander” between 1960 and November 2018. Articles, including case reports/series, clinical trials, letters to the editor, and retrospective series, were taken into consideration if they met the following criteria: patients had received single or multiple fractions of RT in combination with immunotherapy and following RT, anatomic and/or metabolic regression at a non-irradiated tumor site was documented.

Articles were disregarded if a concurrent cytotoxic treatment with RT was given. Studies including subsequent systemic treatments other than immunotherapy after RT were considered ineligible unless an abscopal response was assessed before the subsequent systemic treatment was given.

Cases were evaluated for the dose and fractionation of RT, sequencing, the duration of immunotherapy, and time to abscopal effect. The biologically effective dose (BED) was calculated for each case separately according to the following formula: BED=nd [1+ d/(α/β)], where n is the number of fractions; d is the dose per fraction; and α and β are constants that represent lethal and sub-lethal damage, respectively. The α/β ratio was assumed to be 10 Gy, as generally adopted for rapidly proliferating cells [5-6].

The time to an abscopal effect was defined as that from the end of RT to the documentation of any response in a nonirradiated site.

## Review

Results of the search 

An abscopal effect after RT with or without immunotherapy was reported in 94 cases in 52 articles, including one proof-of-principle trial, one phase I trial, one retrospective series, one letter to the editor, and 48 case reports with one or more cases presented [7-10]. Of the 94 patients, 47 were treated with RT only and reported between 1969 and 2018. However, 47 cases were treated with a combination of RT and immunotherapy, and the reported timeline for these cases was six years (2012–2018).

Using the described selection criteria, one proof-of-principle trial with 11 cases were considered to be ineligible because concurrent chemotherapy was used [7]. One phase I trial with five cases, one letter to the editor and two case reports with four cases were considered ineligible because of a lack of information about the RT dose, fractionation, and site of RT [8,10-12]. One retrospective database analysis with 11 cases, which had incomplete data on the patient characteristics, and the period to an abscopal effect was still included in considering tumor histology and RT parameters [9].

Finally, a total of 24 cases in 15 reports that demonstrated an abscopal effect were reviewed. Results are summarized in Table 1.

**Table 1:** Patient and treatment characteristics reported in clinical cases of abscopal effect after radiotherapy and immunotherapy ca: cancer, Tx: treatment, CT: chemotherapy, RT: radiotherapy, INF: interferon, stem cell trans: stem cell transplantation, TKI: tyrosine kinase inhibitors, met: metastasis, WBRT: whole brain radiotherapy, SRS: stereotactic radiosurgery, Ln: lymph node, Gy: gray, no.: number; fr: fractions, BCG: bacillus Calmette-Guerin vaccine, GM-CSF: granulocyte-macrophage colony-stimulating factor, DC: dendritic cell, CIK: cytokine-induced killer cell, mo: months, PR: partial response, CR: complete response. SD: stable disease.

Reference	Histology	Age	Sex	Prior Tx	RT site	Total dose (Gy)	No. of fr	Dose/fr (Gy)	Immun modulation Tx	Time to abscopal effect (mo)	Response	Site for abscopal effect
Postow et al. [13]	Melanoma	33	F	CT-surgery	Paraspinal mass	28.5	3	9.5	Ipilimumab	4	PR	Lung+spleen met
Hiniker et al. [14]	Melanoma	57	M	Surgery-RT-INF	Hepatic met (2 lesions)	54	3	18	Ipilimumab	4	CR	Liver met
Golden et al. [15]	NSCLC-adenoca	64	M	CT-RT	Hepatic met	30	5	6	Ipilimumab	2,5	PR	Lung+Liver+Bone metastasis
Grimaldi et al. [9]	Melanoma	n/a	n/a	Systemic tx	Brain (WBRT)	30	10	3	Ipilimumab	n/a	PR	Liver met
	Melanoma	n/a	n/a	Systemic tx	Brain (WBRT)	30	10	3	Ipilimumab	n/a	PR	Liver met
	Melanoma	n/a	n/a	Systemic tx	Chest Wall +right axilla	50	25	2	Ipilimumab	n/a	PR	Liver + cutaneous met
	Melanoma	n/a	n/a	Systemic tx	Right inguinal LN	20	5	4	Ipilimumab	n/a	PR	Gastric+cutaneous+lung+ nodal+abdominal met
	Melanoma	n/a	n/a	Systemic tx	Brain (WBRT)	30	10	3	Ipilimumab	n/a	PR	Liver + over +nodal met
	Melanoma	n/a	n/a	Systemic tx	Brain (WBRT)	30	10	3	Ipilimumab	n/a	PR	Lung+cutaneous+abdominal+nodal met
	Melanoma	n/a	n/a	Systemic tx	Chest Wall	30	10	3	Ipilimumab	n/a	SD	Cutaneous+chest wall + nodal met
	Melanoma	n/a	n/a	Systemic tx	Vertebra	30	10	3	Ipilimumab	n/a	SD	Lung met
	Melanoma	n/a	n/a	Systemic tx	Brain (SRS)	24	1	24	Ipilimumab	n/a	PR	Cutaneous met
	Melanoma	n/a	n/a	Systemic tx	Brain (SRS)	20	1	20	Ipilimumab	n/a	PR	Liver met
	Melanoma	n/a	n/a	Systemic tx	Brain (SRS)	24	1	24	Ipilimumab	n/a	PR	Lung met
Kodama et al. [12]	NSCLC-adenoca	74	M	Surgery-KT	Supraclavicular fossa	48 (+10Gy/fr boost)	24	2	BCG	6 (PR) 8 (CR)	CR	Nodal met
Michot et al. [16]	Hodgkin Lymphoma	33	M	CT-stem cell trans	Mediastinal LN	30	10	3	Pembrolizumab	2	CR	Subdiagraphmatic LNG
Shi et al. [17]	Pancreatic c	67	F	CT	Pancreas (primary)	45	15	3	GM-CSF	1	PR	Liver met
Cong et al. [18]	NSCLC-adenosquamous ca	64	F	CT-TKI	Lung met	37.5	5	7.5	DC-CIK	10	CR	Lung met
La Plant et al. [19]	RCC	24	F	RT-TKI-CT	Bone met	27	3	9	Ipilimumab	7	CR	Lung met +nodal met
Sharabi et al. [20]	Cervical ca-large cell neuroendocrine ca	48	F	Surgery-CT-RT	Abdominal mass	20	4	5	Nivolumab	5	PR (95%)	Hepatic met+pelvic mass+ nodal met
Sato et al. [21]	Gastric cancer	54	M	Surgery-CT	Stomach (primary)	48	24	2	T cell +DC therapy	2	PR	Peritoneal met
Zhao et al. [22]	Esophagus	65	M	CT	Retroperitoneal LN	42	6	7	Pembrolizumab	2	CR	Nodal met
Brtitschgi et al. [23]	NSCLC-adenoca	47	M	CT-RT-TKI	Abdominal LN	18	3	6	Nivolumab	3	CR	Nodal met
Tsui et al. [24]	Mucosal melanoma	65	F	RT-epacadost	Neck mass	24	3	8	Nivolumab	First fraction	PR	Lung met

Patient and Tumor Characteristics

Thirteen cases were evaluated for patient characteristics. Patients’ ages ranged from 24 to 74 years with a median age of 57 years. Cases included seven male patients and six female patients. Non-small cell lung cancer (three adenocarcinomas and one adenosquamous carcinoma) was the most frequent histology with four cases followed by three cases of melanoma (one mucosal and two cutaneous). Other cases retrieved were of Hodgkin's lymphoma, esophageal cancer, gastric cancer, pancreatic cancer, cervical cancer with large cell neuroendocrine carcinoma histology, and renal cell cancer. 

All 11 cases presented in the retrospective analysis had malignant melanoma, bringing the number of melanoma cases to a total of 15.

All the patients had undergone prior treatments. Among the reported data, 12 patients had received systemic therapies. Four patients had undergone surgery either for resection of the primary site or metastasis. Five patients had had prior RT exposure as part of their definitive or palliative treatment. One of the patients also had received a stem cell transplant.

Thirteen case reports detailed the period between prior treatments and immunotherapy. Most patients started the immunotherapy immediately after initial systemic treatment: two patients two months after, one patient seven months after, one patient nine months after, and one patient had started after two years.

RT Parameters

Twenty-four patients had RT at 25 sites in total, one patient had RT at two sites.

RT doses and fractionation varied widely, with a median total dose of 30 Gy (18 Gy–58 Gy) in a median number of 5.5 fractions (1–25). Doses per fraction ranged from 2 Gy to 24 Gy (median: 4.5 Gy). BED 10 was calculated to be median 49.65 Gy (28–151 Gy).

Fractionation schemes were evaluated in four groups:

1) Conventional fractionation (1.8–2 Gy/fr): three patients (one patient had conventional fractionation with a boost of 10 Gy/fr).

2) Moderate hypofractionation (3–6 Gy/fr): 12 patients

3) Hypofractionation (7–10 Gy/fr): 5 patients

4) Ablative doses (>12 Gy): four patients (one patient had RT at two different sites with an ablative dose of 18 Gy/fr)

Two patients were irradiated at the primary tumor site, and 18 at a distant organ metastasis and four at a nodal site.

The target volume was not reported. RT technique was reported for 14 patients. Thirteen of the patients were treated with stereotactic body radiotherapy/stereotactic radiosurgery (SBRT/SRS) or intensity-modulated radiotherapy (IMRT) and only one patient was treated with three-dimensional conformal radiotherapy (3DCRT).

Sequencing of Therapies

Most cases (23 of 24) of abscopal response occurred in patients who received RT concurrent with or immediately after immunotherapy. Twenty-one patients were initially given immunotherapy and required RT because of disease progression. RT was part of the initial treatment in two patients. One of these patients had a granulocyte-macrophage colony-stimulating factor (GM-CSF) concurrently, and the other patient subsequently received Bacillus Calmette-Guerin (BCG) vaccine [12,17].

Evaluation of Abscopal Response

Among 24 patients; seven demonstrated complete response (CR), 15 patients had partial response (PR), and two patients had stable disease (SD). The rate of regression was reported in only two cases. Sharabi et al. [20] reported the rate of regression as 95% and Sato et al. as unmeasurable [21]. Most patients that were classified as PR were reported to have a minimal disease.

Discussion of the results

A total of 24 reported cases demonstrating the abscopal effect were systematically reviewed in this article, and these are summarized in Table 1. Although preclinical studies support this phenomenon, the rarity of reported clinical cases on the abscopal effect suggests that there is a high threshold for immune system activation translate into a clinically meaningful response [25]. The abscopal effect has received greater attention with the more frequent use of immunotherapies. Our search between 1960 and 2018 revealed 94 cases describing the abscopal effect. Strikingly, of the 94 patients, 47 were treated only with RT and reported between 1969 and 2018. However, 47 patients were treated with a combination of RT and immunotherapy, and the timeline for these reported cases was six years (2012-2018).

Further review of the reports of the 47 patients that received RT and immunotherapy, revealed some conflicting points. The definition of the abscopal effect varied among the reports. Some reports accepted concurrent chemotherapy [7]. For our selection criteria, articles were disregarded if concurrent systemic treatment with RT was given to ensure demonstration of a clear interaction between RT and immunotherapy. Similarly, articles with subsequent systemic treatment other than immunotherapy after RT were considered ineligible unless an abscopal response was assessed before the subsequent systemic treatment was given [12].

In their proof of principle study where they evaluated the addition of GM-CSF, Golden et al. reported that patients with an abscopal response had a better outcome [7]. This data rendered the abscopal effect more attractive yet the question of how to increase the frequency of clinically significant remains to be answered. Kang et al. summarized four main issues that should be resolved when designing prospective trials, including patient selection, RT parameters, sequencing of therapies, and endpoint selection [4]. These points also formed the basis of the present analysis.

Patient Selection

In all of the cases analyzed, the patients had undergone prior treatment. Most patients had switched to immunotherapy either because of their low tolerance to chemotherapy or owing to progressive disease. It is known that patient selection is critical in the use of RT and immunotherapies. Factors such as the degree of myelosuppression, overall tumor burden, neutrophil-to-lymphocyte ratio, and prior exposure to RT and chemotherapy should be taken into consideration [4,7]. Only two case report provided gave detailed information on the change in blood counts [16,20]. As the depletion of immune cells is expected to decrease the immune response, patients with decreased lymphocyte counts due to cytotoxic chemotherapy and marrow infiltration by tumor are likely to be poor responders to treatment. In the included reports, most patients subsequently started immunotherapy after the failure of prior treatments.

Historically, several tumor types, including melanoma, renal cell carcinoma, and lymphoma, are thought to be immunogenic [13,26-27]. The abscopal effect was mostly expected during the treatment of these tumors. In our review, remarkable abscopal responses were also seen in other histologic types, such as gastric, esophageal, or pancreatic cancers [17,21-22]. This observation is consistent with preclinical data, suggesting that the combination of RT and immunotherapy induces effective immune responses to poorly immunogenic carcinomas [28-29].

RT Parameters

Optimal dose and fractionation have been controversial since their importance was highlighted in preclinical studies [29-30]. It was shown that a single 20 Gy dose of radiation is less effective to generate an abscopal effect than regimens of 24 Gy in three fractions or 30 Gy in five fractions during the immune checkpoint blockade therapy [27]. On the contrary, some researchers have questioned the need for a fractionated regimen and, instead, have proposed the use of single ablative doses as a better approach to induce abscopal effects. In a phase I study, Maity et al. tested hypofractionation (24 Gy/3fr) and single fraction (17 Gy/fr) doses and demonstrated an abscopal response under both schemes [8]. Moreover, in the recently published trial by Antonia et al., patients with locally advanced lung cancer, including those with low programmed-cell death ligand-1 (PDL-1) levels, were randomized to receive immunotherapy with an anti-PD-L1 antibody or placebo after a conventionally fractionated round of chemoradiotherapy. The immunotherapy arm had much better progression-free survival than the placebo arm [31]. Considering the low threshold of PDL-1 levels, the increment was attributed to the interaction between RT and immunotherapy. This highlights the question of whether conventionally fractionated RT is suitable for creating an abscopal effect.

In this review, RT doses and fractionation varied widely across the cases analyzed. Fractionation schemes were evaluated in four groups: 1) conventional fractionation 2) moderate hypofractionation (3-6 Gy/fr) 3) hypofractionation (7-10 Gy/fr) 4) ablative doses (>12 Gy). Although most patients were treated with moderate hypofractionation, it should be noted that in general doses of 3-5 Gy/fr are the preferred scheme for metastatic disease sites. 

Other than fraction dose, BED10 is considered an important parameter for the abscopal effect. In their meta-analysis of preclinic models, Marconi et al., showed that the occurrence rate of abscopal effects increases in parallel with BED. Mainly, the probability of revealing abscopal effects was 50% when a BED of 60 Gy was reached [32]. In our analysis BED ranged widely from 28 to 151.2 Gy and only one-third of patients reached BED10 of 60Gy or higher.

The use of large RT fields has been claimed to reduce the availability of effector and memory cells. Because circulating lymphocytes are highly sensitive to RT, with a D90 of 0.5 Gy [33], larger treatment fields are viewed as a shortcoming in obtaining a clinically compelling response. Other conformal techniques, such as SBRT or IMRT have become predominant as they are considered to reduce the treated volume. Unfortunately, field sizes were not reported in most of the included cases. Among the 14 patients where RT technique was noted, 13 patients were treated with SBRT/SRS or IMRT, and only one was treated with 3DCRT.

Sequencing of Therapies

The optimal sequencing of RT with immunotherapy likely depends on the type of treatment used and mode of action. Preclinical models support the use of prior and /or concomitant administration of immunotherapy more than initializing of RT, followed by immunotherapy [4]. Similarly, the majority of currently ongoing trials employ concurrent use. By contrast, Schmidberger et al. [34] pointed out in their retrospective analysis of 41 melanoma patients with brain metastasis that applying RT before immunotherapies prolonged survival. They showed that patients treated with ipilimumab after RT had a median survival of 11 months, compared with three months for the patients who received ipilimumab prior to RT.

In our review, it is observed that most of the reports (23 out of 24), abscopal response occurred in patients who received RT concurrent with, or immediately after immunotherapy. In most of the reports RT was preferred for symptom palliation and in only one case report RT was applied not because of progression but with the intent to boost the immune response [19]. Gathering the preclinical data and clinical reports together, an intriguing question would be whether we would observe more cases of abscopal effect when RT is viewed as part of planned therapies either prior or subsequent to immunotherapy.

Endpoint (Abscopal Effect) Evaluation

Endpoint selection for trials with immunotherapy is a point of a discussion [4]. One part of this discussion is evaluation of the abscopal effect. Despite increased interest in the abscopal effect, no consensus definition exists. For example, Formenti and others have defined an abscopal response as a 30% reduction in the size of one nonirradiated metastasis [1]. Moreover, Luke et al.defined abscopal response as the sum of the largest diameter for all of the nonirradiated Response Evaluation Criteria in Solid Tumors (RECIST) target metastases [35].

None of the reports elucidate their definition of the abscopal effect. A vague response assessment, such as minimal disease is implied in most case reports. Only two reports with PR clarified the response rate [20-21].

When an abscopal effect can be expected is also unclear. In reviewing the cases, we found that time to documented abscopal response was available in only 13 case reports. For these 13 patients, the time ranged widely from less than one month to 12 months. This variability probably stems from the arbitrary time points and methods of tumor response evaluation. Still, it is noteworthy that 10 of the 13 patients demonstrated abscopal effects in less than six months. 

Future Directions

The increased number of cases reported with abscopal effect after the use of immunotherapy is striking. Moreover, with the help of immune modulation, RT should be regarded as an effective modality to enhance the systemic immune response. However, there is no doubt that we are still in search of optimal ways to incorporate RT in combination treatments with respect to dose and the fractionation and sequencing of therapies. Comparative regimens to establish ideal RT parameters should be tested in a clinical setting.

Another area that could trigger interest would be integrating biomarkers to objectively assess treatment effects on the immune system.

To achieve these goals, RT should be considered not only for palliation but also with the aim to acquire synergy and, eventually, generate an abscopal effect.

## Conclusions

In summary, this review revealed that the abscopal effect may be observed at all ages and across a variety of tumor types but mostly in immunogenic tumors, as can be expected. Different RT regimens and techniques, particularly conformal techniques such as IMRT or SBRT/SRS, could have an important role. It is clear that optimized doses and fractionation, as well as patient selection, should be considered when incorporating RT with immunotherapies to more frequently obtain clinically significant responses.
